# Identification, genetic testing, and management of hereditary melanoma

**DOI:** 10.1007/s10555-017-9661-5

**Published:** 2017-03-10

**Authors:** Sancy A. Leachman, Olivia M. Lucero, Jone E. Sampson, Pamela Cassidy, William Bruno, Paola Queirolo, Paola Ghiorzo

**Affiliations:** 1 Department of Dermatology and Knight Cancer Institute, Oregon Health & Science University, Portland, OR USA; 2 Department of Molecular and Medical Genetics, Oregon Health & Science University, Portland, OR USA; 3 Department of Internal Medicine and Medical Specialties, University of Genoa and Genetics of Rare Cancers, IRCCS AOU San Martino-IST, Genoa, Italy; 4 Department of Medical Oncology, IRCCS AOU San Martino-IST, Genoa, Italy

**Keywords:** Melanoma, Genetic testing, Inherited cancer risk, Gene panel sequencing, Genetic syndromes

## Abstract

Several distinct melanoma syndromes have been defined, and genetic tests are available for the associated causative genes. Guidelines for melanoma genetic testing have been published as an informal “rule of twos and threes,” but these guidelines apply to *CDKN2A* testing and are not intended for the more recently described non-*CDKN2A* melanoma syndromes. In order to develop an approach for the full spectrum of hereditary melanoma patients, we have separated melanoma syndromes into two types: “melanoma dominant” and “melanoma subordinate.” Syndromes in which melanoma is a predominant cancer type are considered melanoma dominant, although other cancers, such as mesothelioma or pancreatic cancers, may also be observed. These syndromes are associated with defects in *CDKN2A*, *CDK4*, *BAP1*, *MITF*, and *POT1*. Melanoma-subordinate syndromes have an increased but lower risk of melanoma than that of other cancer(s) seen in the syndrome, such as breast and ovarian cancer or Cowden syndrome. Many of these melanoma-subordinate syndromes are associated with well-established predisposition genes (e.g., *BRCA1/2*, *PTEN*). It is likely that these predisposition genes are responsible for the increased susceptibility to melanoma as well but with lower penetrance than that observed for the dominant cancer(s) in those syndromes. In this review, we describe our extension of the “rule of twos and threes” for melanoma genetic testing. This algorithm incorporates an understanding of the spectrum of cancers and genes seen in association with melanoma to create a more comprehensive and tailored approach to genetic testing.

## Introduction

A small portion of melanomas (approximately 5–12%) occurs in patients with a strong family history of melanoma [1, 2]. About 45% of these familial melanomas have been attributed to inheritance of a mutation in a highly penetrant predisposition gene [3]. The 55% “missing inheritance” is likely due to the inheritance of lower-penetrance predisposition genes in combination with inheritance of polymorphisms and/or shared environmental exposures that predispose toward melanoma, culminating in a familial pattern of melanoma inheritance. In addition to this “melanoma-dominant” pattern of inheritance, melanoma can also be a “subordinate” cancer in the context of other cancer syndromes. For example, family members with Cowden syndrome, Li Fraumeni syndrome, and some cancer-specific syndromes such as breast and ovarian cancer syndrome have a poorly-defined but elevated risk of melanoma. The genes associated with these other types of cancer syndromes (e.g., *phosphatase and tensin homolog* (*PTEN)*, *tumor protein p53* (*TP53*), *breast cancer 1 and 2* (*BRCA1/2*), *xeroderma pigmentosum* (*XP A-G*)) are therefore acting as a lower-penetrance melanoma gene in the context of that syndrome [1]. Within the melanoma-dominant syndromes caused by *cyclin-dependent kinase inhibitor 2A* (*CDKN2A*), and *BRCA1-associated protein 1* (*BAP1*), pathogenic mutations in these genes cause increased incidence of other cancer types (e.g., pancreatic cancer, neurological tumors, renal cell carcinoma, mesothelioma) but with lower penetrance than melanoma [2].

As cancer predisposition syndromes and their associated genes are clarified, significant, previously unappreciated overlap is being identified: melanoma genes can be associated with other cancer types and other cancer predisposition genes can increase the risk of melanoma.

This overlap has major implications for genetic testing strategies. Especially in an era when reasonably priced multi-gene panel tests are available, it no longer seems appropriate to offer genetic testing to high-risk melanoma patients for only *CDKN2A* when other genes are known to cause melanoma-dominant syndromes. In addition, there should likely be special consideration given to families that fail to meet the genetic testing criteria for a melanoma-dominant syndrome yet also have other cancers suggestive of a “melanoma-subordinate” cancer syndrome. For example, consider a family with first- and second-degree relatives that have had two melanomas, a breast cancer at age 51 and a prostate cancer at age 42. Although this scenario does not meet formal criteria for *CDKN2A*, *BRCA*, or *TP53* genetic testing, failure to diagnose an actionable mutation in this family could lead to inadequate cancer screening. In addition, because different subspecialty providers often care for family members with different cancer types, the benefit of additional cancer screening beyond that recommended for a single cancer might not be recognized without identification of a unifying genetic cancer predisposition.

Genetic testing of appropriate individuals and the tailored follow-up recommendations that ensue can improve early detection, reduce mortality, and enhance compliance with prevention recommendations [4, 5]. Furthermore, if the highest-risk individuals are selectively screened and treated before expensive systemic therapies are required, costs can be reduced and productive years of life can be increased.

Numerous panel tests are now commercially available allowing for single pass screening of tumor susceptibility genes at about the same cost that was previously paid for single-gene testing, making panel testing an attractive option for melanoma families. Given this and the increasing recognition of cancer syndromes that include melanoma, we offer a comprehensive, tailored cancer gene assessment tool for families with a hereditary pattern of cancer that includes melanoma. This article will focus on how to utilize risk assessment strategies to identify appropriate candidates for genetic testing, identify genes for a tailored panel test, interpret genetic test results, and utilize results to make practical clinical management recommendations. It will also highlight how improved genetic testing technology can be applied now, with rational suggestions for keeping pace with these advances in clinical practice. By taking this approach, it is possible to incorporate more comprehensive technology into patient care (without substantially increasing cost), advance our understanding of the spectrum of cancers observed in association with predisposition genes, and provide appropriate advice to patients that can be augmented as medical knowledge increases.

## Approach to minimize the caveats of panel testing

In addition to benefits of melanoma genetic testing, there are also costs associated with genetic testing and heightened surveillance, including increased frequency of biopsies and other procedures. For this reason, it is critical that individuals are pre-screened to identify those with a reasonable probability of carrying an actionable mutation. It is equally important to provide accurate risk statistics to patients who undergo genetic testing, and this includes acknowledgment that some data is incomplete and thus no change in management is yet recommended. It is essential that the counselor and patients feel comfortable with the possibility of getting unclear results returned to them, and this should be included as part of the informed consent process.

Another caveat of panel testing is that follow-up and management recommendations for some gene mutations are not well developed. To mitigate this effect, our practice is to report only genes with a strong clinical association. Genes that have limited evidence should be used for research purposes only. Research-related results should be reported after more definitive guidance is available, similar to reports for variants of uncertain significance. The reportable genes, as well as some of their corresponding cancers, have been listed in Table 1.

**Table 1:** Reportable genes and some of their associated cancers

Gene symbol	Cutaneous melanoma	Pancreatic cancer	Breast cancer	Prostate cancer	Colon cancer	Ovarian cancer
*APC*		• [57]			• [56]	
*ATM*		• [60]	• [61]			
*AXIN2*					• [62]	
*BAP1*	• [34]					
*BARD1*			• [63]			
*BMPR1A*		• [64]			• [64]	
*BRCA1*		• [60]	• [53]	• [54]		• [53]
*BRCA2*		• [60]	• [53]	• [54]		• [53]
*BRIP1*			• [67]			• [67]
*CDH1*			• [68]		• [69]	
*CDK4*	• [23]	• [23]				
*CDKN2A*	• [2]	• [60]				
*CHEK2*			• [70]	• [71]	• [72]	
*DICER1*						• [73]
*EPCAM*		• [74]			• [74]	• [74]
*FANCC*						
*GREM1*					• [75]	
*HOXB13*				• [76]		
*MEN1*		• [77]				
*MITF*	• [35]					
*MLH1*		• [60]		• [78]	• [79]	• [80]
*MSH2*		• [60]		• [78]	• [79]	• [80]
*MSH6*		• [60]		• [78]	• [79]	• [80]
*MUTYH*					• [81]	
*NBN*			• [88]			
*NF1*			• [84]			
*PALB2*		• [60]	• [86]			
*PMS2*		• [60]		• [78]	• [79]	• [79]
*POLD1*					• [87]	
*POLE*					• [87]	
*POT1*	• [27]					
*PTEN*			• [84]		• [49]	• [84]
*RAD50*			• [88]			• [89]
*RAD51C*						• [90]
*RAD51D*						• [91]
*SMAD4*		• [64]			• [64]	
*SMARCA4*						• [92]
*STK11*		• [60]	• [93]		• [93]	• [93]
*TP53*		• [94]	• [95]	• [94]	• [95]	• [95]
*TSC1*		• [96]				
*TSC2*		• [96]				
*VHL*		• [97]				

References in brackets

By collaborating with our patients to perform tests that are currently in the research realm and counseling them on the uncertain significance of the results as our knowledge of genotype-phenotype relationships improves, we can simultaneously move the field forward faster both clinically and scientifically. It is important to note that if mutations that have only preliminary evidence for pathogenicity are included in the testing process, it will require additional genetic counseling to assure that the patient understands the equivocal nature of mutations found in these genes, similar to the discussion regarding variants of unknown significance (VUS). This additional counseling effort may not be possible and/or advisable in some situations or for some patients. In this case, it may be best for those providers and patients to utilize a single-gene or panel test that does not contain research or preliminary genes, at the discretion of the provider.

## Background: melanoma predisposition genes

As stated above, several melanoma predisposition genes have been identified to date. The most common is the *CDKN2*A gene locus, which is involved in approximately 20–40% of large, high-risk families [6, 7]. The *CDKN2A* gene locus encodes two melanoma predisposition genes with different functions: *CDKN2A/p16*, a tumor suppressor that imposes control through the retinoblastoma (Rb) pathway [8, 9], and *CDKN2A/ARF*, another tumor suppressor that functions through the p53 pathway [10]. Families that carry a pathogenic mutation in *CDKN2A* have an increased risk for melanoma, pancreatic cancer, and perhaps neurological tumors like astrocytoma [6, 11]. Individuals in these families frequently, but not always, have a large number of atypical moles. A substantially increased number of atypical moles in the setting of a *CDKN2A* mutation has been termed familial atypical mole and malignant melanoma syndrome (FAMMM), although it is also called the familial melanoma and pancreatic cancer syndrome (FMPC) or the familial atypical multiple mole melanoma-pancreatic carcinoma syndrome (FAMMPC), because of the increased risk of pancreatic cancer [12–18]. Clinical genetic testing for *CDKN2A* mutations has been widely available since the mid-2000s, and guidelines for use of this test were published in 2009 and are detailed below [19].

Although *CDKN2A/p16* is the most common and best characterized melanoma-dominant predisposition gene, several additional, less common predisposition genes are associated with heritable risk for melanoma and other cancers including *alternate reading frame* (*CDKN2A/ARF*) [20], *cyclin-dependent kinase 4* (*CDK4*) [21–23], *telomerase reverse transcriptase* (*TERT*) [24, 25], *protection of telomeres 1* (*POT1*) [26, 27], *adrenocortical dysplasia* (*ACD*) [28], *telomeric repeat-binding factor-2* (*TERF2*) *interacting protein* (*TERF2IP*) [28], *breast cancer gene 1* (*BRCA1*)*-associated protein 1* (*BAP1*) [29–34], and *microphthalmia-associated transcription factor* (*MITF*) [35–39]. Most recently, the tumor suppressor *BAP1* has been identified as the causal gene in a tumor predisposition syndrome including, among others, atypical Spitz tumors, cutaneous and uveal melanoma, mesothelioma, and clear cell renal carcinoma. Deleterious mutations in these genes lead to a disproportionately high risk of melanoma development relative to other cancers.

Clear guidelines for genetic testing of these non-*CDKN2A* genes have not been published, despite the availability of these tests in several clinically certified laboratories. Informally and in research protocols, family members that meet the criteria for *CDKN2A/p16* testing have been tested at the entire locus, including the *CDKN2A/ARF* component and promoter regions. Families shown to be mutation negative at the *CDKN2A* locus are sometimes tested reflexively for the most common *CDK4* mutation site, Arg24. This *CDKN2A*/p16 binding site (Arg24) is required for *CDK4* inhibition by *CDKN2A*/p16 and mutation transforms the protein into an unregulated oncoprotein [40], making single site testing of this codon a reasonable consideration.

Melanoma is also observed at higher-than-expected rates in other hereditary cancer syndromes arising from germline mutations in tumor susceptibility genes (melanoma-subordinate syndromes). More specifically, increased risk for melanoma is seen in xeroderma pigmentosum (multiple *XP* genes) [41], Cowden syndrome (*PTEN* mutations) [42], Li Fraumeni syndrome (*TP53* mutations) [43], and possibly others. However, guidelines for melanoma screening and genetic testing are unclear in most of these syndromes. When features of these syndromes are identified, it is important to include counseling about melanoma prevention and follow-up recommendations as part of their genetic counseling session. Furthermore, use of genetic panel testing for a variety of syndromes, including those with a predominance of melanoma, will begin to refine the constellation of cancers seen in association with various mutations.

## Identification and selection of melanoma genetic testing candidates

In 2009, international guidelines were published suggesting that individuals with an estimated 10% or greater pre-test probability of carrying a mutation in *CDKN2A* should be referred for genetic counseling [19]. However, accurate estimates of pre-test probabilities of mutation carriage were complicated by the interdependence upon ethnicity and geography that underlie a general population’s risk for melanoma. These guidelines suggest that candidacy for counseling and *CDKN2A* testing be based on the number of (1) invasive primary melanomas in the identified patient (proband), (2) invasive melanomas in blood relatives, and (3) the numbers of pancreatic cancers in the proband or blood relatives. The minimum number of cancers required to establish candidacy is based on the baseline population rates of the family’s place of residence. This rationale posits that individuals with a high genetic risk for melanoma would have substantially more melanomas (or melanomas plus pancreatic cancer) than the general population in their area. For example, in areas of intermediate-to-high population risk of melanoma such as the USA or Northern Europe (with estimated, age-standardized rates ≥10 per 100,000), individuals or families with a total of three or more primary melanomas (or combinations of melanoma and pancreatic cancer) would be referred for genetic counseling. However, in areas with lower melanoma incidence (rate <10 per 100,000), such as Italy, Spain, or France, development of two or more melanomas (or melanoma and pancreatic cancer combinations) in an individual or family would be referred for counseling. Use of this “rule of twos or threes” criteria for melanoma genetic testing leads to approximately 10% positive results for *CDKN2A* mutation. It is important these guidelines not be viewed as absolute “rules” that replace clinical judgment in individual circumstances. For instance, it is unclear how environment, other prognostic risk factors such as the hair, eye, and skin color, and/or ethnicity contribute to the overall risk in individuals with *CDKN2A* (or other melanoma gene) mutations. Furthermore, in Italy, it has been determined that *in situ* melanomas can be included in the “rule of two” criteria applied to the Italian population [44, 45], while in the USA, this data is based on invasive melanomas and individuals with melanoma *in situ* should be evaluated on an individual basis.

As described above, many additional cancer susceptibility genes have been identified that are associated with an increased risk of melanoma (melanoma-subordinate syndromes) and other cancers. In this paper, we update the “rule of twos and threes” that takes other non-melanoma cancer types into consideration. To accomplish this task, we have created a simplified scoring system that identifies individuals or families that have a pattern of cancer development suspicious for germline inheritance of a pathogenic mutation. In this assessment tool, a score of 3 is required as the threshold with which to proceed with genetic testing, effectively creating a universal “rule of threes.” To create this tool, we have incorporated criteria from several sources. First, we have accounted for high and low melanoma geographic areas by designating one point in moderate or high-incidence areas and 1.5 points in low-incidence areas. Second, we have included guidelines used in cancers other than melanoma that, in their own right, would fulfill criteria for genetic testing but also have a family member with melanoma. These findings count as one point; however, it is important to note that occurrence of these findings in the absence of melanoma would prompt consideration of genetic testing. Third, we account for clinical findings consistent with the melanoma-dominant tumor syndrome caused by a *BAP1* mutation. Lastly, we have accounted for a high incidence of cancers in a single individual or family that do not otherwise meet criteria for genetic testing. The original “rule of twos and threes” was based on finding criteria that would provide at least a 10% pre-test probability of finding an actionable *CDKN2A* mutation. Ideally, we would apply this same pre-test probability for every permutation of melanoma and other melanoma-subordinate syndrome cancer types. However, this level of data does not yet exist. In the absence of hard data, our institutions have felt that it is not appropriate to consider all other cancers equal with respect to fulfilling the rule of three. There are three cancers in melanoma-subordinate syndromes with a greater lifetime probability of developing invasive cancer than melanoma itself (approximately ≥4–5%), including prostate, breast, and colon. In the case of these cancers, assuming they do not fulfill other requirements for consideration as a genetically associated cancer, our centers “count” two of these cancers as one point in meeting the “rule of threes” criteria. For example, if an individual in the USA with melanoma has one second-degree blood relative with melanoma and one with breast cancer, then she would not fulfill the criteria unless the individual with breast cancer was of a younger age, of male gender, or otherwise highly suspicious for having a genetic cause. However, if there were two breast cancer occurrences in addition to the two melanoma cases, then the “rule of threes” would apply (Table 2).

**Table 2:** Melanoma cancer syndrome assessment tool

Cancer type	Criteria	Points per occurrence
Melanoma	Occurrence in melanoma proband, first- or second-degree relative^a^	1 or 1.5^b^
Astrocytoma^c^	Occurrence in melanoma proband, first- or second-degree relative	1.5
Breast	Occurrence in proband, first- or second-degree relative under 45 years of age	1^d^
	Occurrence of bilateral or triple negative breast cancer in proband, first- or second-degree relative	1^d^
	Occurrence in male gender	1^d^
Colon	Occurrence in proband or first-degree relative that occurred under 50 years of age	1^d^
	Proband has had more than five adenomatous polyps occurring under 50 years of age	1^d^
Ovarian	Occurrence in proband, first- or second-degree relative	1
Pancreatic^c^	Occurrence in proband, first- or second-degree relative	1.5
Prostate	Proband has had metastatic prostate cancer and/or had a Gleason score >7 at diagnosis	1^d^
High frequency	At least two occurrences of breast, colon, or prostate cancer in melanoma proband, first- or second-degree blood relatives that do not meet the criteria above	1
*BAP1* cancer syndrome	Occurrence in proband or first-degree relative of uveal melanoma, paraganglioma, mesothelioma, atypical Spitz tumors or clear cell renal carcinoma	1.5/cancer type
Perform genetic testing		3 or more

^a^First-degree relatives include parents, siblings, and children; second-degree relatives are blood relatives that include grandparents, grandchildren, aunts, uncles, nephews, nieces, or half-siblings

^b^1 point in moderate or high melanoma incidence areas and 1.5 points in low-incidence areas. Regions with an estimated, age-standardized incidence rate of <10 per 100,000 is considered low incidence. See GLOBOCAN online for current rates

^c^Pancreatic cancer and astrocytoma are scored 1.5 due to increased incidence in melanoma-dominant syndromes caused by a *CDKN2A* mutation

^d^The criteria listed suggest a hereditary pattern that may fulfill standard criteria for single-gene or cancer-specific panels without association with melanoma. Anyone or any family with these findings should be considered for genetic testing regardless of their melanoma status. However, if the criteria are met in the context of melanoma, we test additionally for melanoma genes

## Choosing the appropriate family member for genetic testing

Whenever a high-risk family has been identified, it is important to select an appropriate family member for genetic testing. Ideally, a member of the family that has had cancer should be tested first and, if possible, the youngest member with cancer or the member with the most dramatic hereditary pattern. This maximizes the chances of identifying an actionable mutation, although it is also conceivable that the individual who has been tested has a sporadic cancer in the context of a familial syndrome. For this reason, if the family history is extremely compelling for having a cancer syndrome but the individual that was tested does not carry a mutation, it may be important to test a second individual in the family. It is not generally a good practice to test an unaffected individual in the family because a negative test could reflect the fact that they are a non-carrier in the family, rather than reflecting the fact that the family does not carry a mutation.

## Tailored genetic testing based on family history

**Fig. 1:**
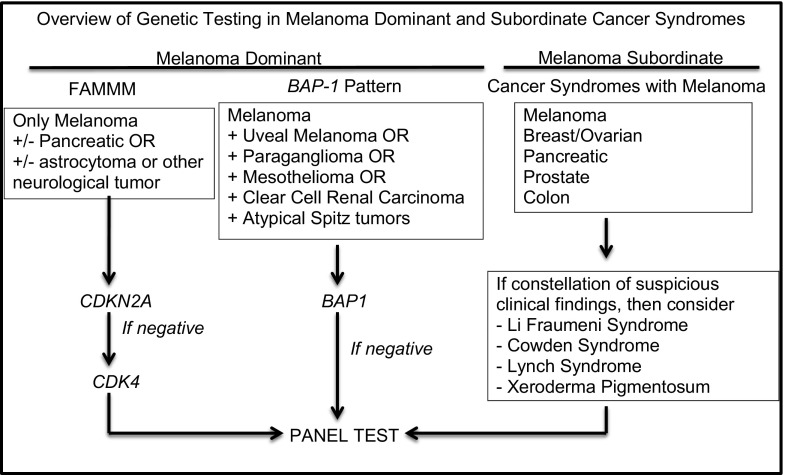
Overview of genetic testing in melanoma-dominant and melanoma-subordinate cancer syndromes. This algorithm details navigation of genetic testing based on family history. It is also an option to proceed directly to panel testing. *FAMMM* familial atypical multiple mole melanoma syndrome. The *left side* of the figure depicts syndromes that contain melanoma as the dominant cancer in the syndrome whereas the *right side* of the figure depicts other cancer syndromes that contain melanoma as a subordinate cancer

In the following section, we detail how our institutions design a personalized genetic panel based on the personal and family history of a suspected hereditary cancer syndrome. This algorithmic approach is summarized in Fig. 1. Once an individual or family has met criteria for genetic testing and has received genetic counseling with informed consent, our centers create a tailored panel test that includes the genes that are most likely to be mutated, based on the other cancer types observed in the pedigree. The rationale for these choices is described below.

### Genetic testing for melanoma-dominant syndromes

**Fig. 2:**
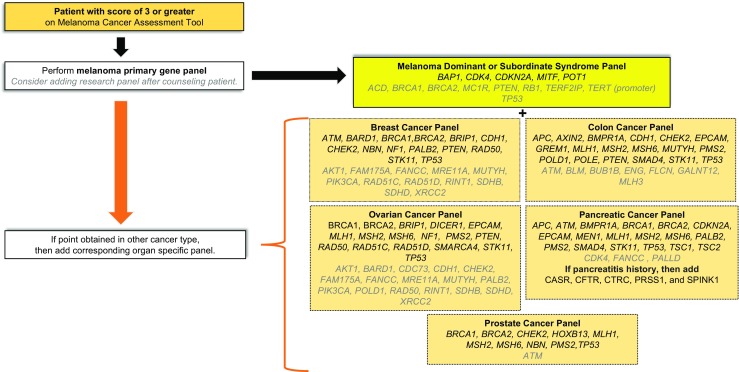
Tailored genetic testing recommendations. If a patient has a score of 3 or greater, then a genetic panel should be tailored to the family history. Genes listed in *gray* should be tested in the research realm after counseling the patient on the risks and benefits

Any patient or family that meets the updated “rule of threes” should be considered a candidate for genetic testing. If melanoma is the only cancer in a pedigree, then to meet the threshold of genetic testing, a pedigree should have three primary melanomas in first- or second-degree relatives in areas with a high melanoma incidence or two primary melanomas in a low-incidence area. This melanoma panel should include *BAP1*, *CDK4*, and *CDKN2A.* Genes for which risk has not been established but for which studies suggest an elevated risk include *MITF* and *POT1* and we recommend including these in the melanoma panel. Genes with a preliminary clinical association include *ACD*, *BRCA1*, *BRCA2*, *MC1R*, *PTEN*, *RB1*, *TERT* (with promoter), *TERF2IP*, and *TP53* which may be included for research purposes (Fig. 2).

Rarely, our institutions may test for *CDKN2A* or *BAP1* as a single test, particularly if other cancers, such as pancreatic cancer or astrocytoma in the family, strongly suggest *CDKN2A* is the offending gene. Similarly, if the family has cutaneous melanomas, uveal melanoma, renal clear call carcinoma, mesothelioma, or paraganglionoma, then a *BAP1* test may be sufficient (Fig. 1). It should also be noted that single-gene testing may be done in cases where a specific mutation in a cancer susceptibility gene has been identified in a pedigree and a relative requests testing to determine carrier status.

### Genetic testing for melanoma-dominant and melanoma-subordinate syndromes that also demonstrate pancreatic cancer

Co-occurrence of pancreatic cancer and melanoma usually occurs in the setting of a *CDKN2A* mutation. However, pancreatic cancer also occurs in melanoma-subordinate syndromes (e.g., *BRCA2*). When pancreatic cancer is present in a family, it is reasonable to assess for genes that carry a high risk of pancreatic cancer. Genes that carry greater than a twofold increase in pancreatic cancer risk include *APC*, *BRCA2*, *CDKN2A*, *EPCAM*, *MLH1*, *MSH2*, and *STK11*. Genes for which risk has not been established but for which studies suggest an elevated risk include *ATM*, *BMPR1A*, *BRCA1*, *MEN1*, *MSH6*, *PALB2*, *PMS2*, *SMAD4*, *TP53*, *TSC1*, and *TSC2* and we recommend including these in the primary panel. Preliminary evidence genes that remain in the research realm include *CDH1*, *CDK4*, *FANCC*, and *PALLD*. Chronic pancreatitis genes may be added if there is suspicious personal or family history of pancreatitis and include *CASR*, *CFTR*, *CTRC*, *PRSS1*, and *SPINK1* (Fig. 2).

### Genetic testing for melanoma-subordinate syndromes with breast cancer

Because breast cancer is relatively common (incidence rate of 12.4% in the USA [46]), we recommend that in order to be included toward fulfillment of the “rule of threes,” breast cancer must have one or more of the following features: (1) one of the melanoma patients or a first- or second-degree relative has had breast cancer under 45 years of age, (2) at least one melanoma patient or first-degree relative has had triple negative or bilateral breast cancer under 60 years of age, or (3) there is a breast cancer occurrence in a male patient, or (4) there are two or more instances of breast cancer in addition to melanoma in the pedigree. In our centers, any of these four breast cancer criteria counts as one point toward the “rule of threes” to fulfill the melanoma testing criteria. It is important to note that if the pedigree was to have more than one of the breast cancer criteria, it would fulfill the criteria for breast cancer genetic testing in its own right, but if a melanoma was also found in the pedigree, it would be reasonable to include melanoma genes in the panel, particularly if traditional breast cancer genes proved to be negative. To account for a high frequency of breast cancer, we also assign a single point to two occurrences of breast cancer at any age in the proband or in first- or second-degree blood relatives that do not otherwise satisfy the above criteria.

If a family is a good candidate for testing based on the occurrence of both melanoma and breast cancer, then both melanoma and breast cancer genes should be tested. In addition to the melanoma primary and research panels, a breast panel would include testing for the following cancer genes that have a minimum twofold increase in breast cancer risk: *ATM*, *BRCA1*, *BRCA2*, *CDH1*, *CHEK2*, *NBN*, *PALB2*, *PTEN*, and *STK11*. Genes that have a strong clinical association but no relative risk statistics to our knowledge include *BARD1*, *BRIP1*, *NF1*, *RAD50*, and *TP53*. In addition to these genes that have been confirmed as actionable, preliminary evidence exists for *AKT1*, *FAM175A*, *FANCC*, *MRE11A*, *MUTYH*, *PIK3CA*, *RAD51C*, *RAD51D*, *RINT1*, *SDHB*, *SDHD*, and *XRCC2* and these could be collected for research purposes (Fig. 2).

### Genetic testing for melanoma-subordinate syndromes with prostate cancer

Prostate cancer is similar to breast cancer with respect to having a relatively high incidence of 14.3% in the general population. For this reason, we feel it is important that in order to count as a point toward the “rule of threes” criteria for genetic testing, one or more of the following prostate cancer criteria be met: (1) one of the melanoma patients has also had metastatic prostate cancer and/or had a Gleason score >7 at diagnosis or (2) at least two family members have had prostate cancer. In addition to having the melanoma panel, the additional reportable prostate gene panel could include *BRCA1*, *BRCA2*, *CHEK2*, *HOXB13*, *MLH1*, *MSH2*, *MSH6*, *NBN*, *PMS2*, and *TP53*. Preliminary evidence genes include *ATM* (Fig. 2)*.*

### Genetic testing for melanoma-subordinate syndromes with colon cancer

Just as with breast and prostate, colon cancer has a higher incidence rate of 4.4 and 4.7% for women and men, respectively [46]. Therefore, to be counted as a point toward fulfillment of the “rule of threes,” the individual or family should have one or more of the following colon cancer findings: (1) one or more of the melanoma patients or one or more of the first-degree relatives should also have had colon cancer under 50 years of age, (2) one of the melanoma patients should have a history of five adenomatous polyps under the age of 50, or (3) at least two occurrences of colon cancer in blood relatives. The primary gene panel for a syndrome with melanoma and colon cancer includes the melanoma genetic panel and genes that have at least a twofold increase in colon cancer risk including *APC*, *BMPR1A*, *CDH1*, *EPCAM*, *GREM1*, *MLH1*, *MSH2*, *MSH6*, *MUTYH*, *PMS2*, *PTEN*, *SMAD4*, *STK11*, and *TP53*. Genes that have a strong clinical association also include *AXIN2*, *CHEK2*, *POLD1*, and *POLE*. The additional preliminary evidence (or research) panel could include *ATM*, *BLM*, *BUB1B*, *ENG*, *FLCN*, *GALNT12*, and *MLH3* (Fig. 2)*.*

### Genetic testing for melanoma-subordinate syndromes with ovarian/uterine cancer

Ovarian and uterine cancers fall into a much lower incidence category than breast, prostate, and colon, showing an incidence of 1.3% for ovarian and 0.8% for uterine. Ovarian and uterine cancers are typically categorized as part of another cancer syndrome, such as the breast and ovarian cancer syndrome or Lynch syndrome. In these cases, certain criteria for these cancers are known to be associated with the hereditary syndromes and are utilized to fulfill the threshold for genetic testing in a family with melanoma. The occurrence of ovarian or uterine cancer in the family counts as one point toward the “rule of threes.” In these cases, both melanoma and ovarian (or uterine) genes should be included in the primary panel test. Genes that have a minimum of a twofold risk increase in ovarian cancer include *BRCA1*, *BRCA2*, *BRIP1*, *DICER1*, *EPCAM*, *MLH1*, *MSH2*, *PMS2*, *PTEN*, *RAD51C*, *RAD51D*, *SMARCA4*, and *STK11*. Genes with a strong association but no relative risk calculations include *MSH6*, *NF1*, *RAD50*, and *TP53*. Given the increased incidence of uterine cancer in Lynch syndrome, melanoma families with uterine cancer should have the addition of colon cancer-associated genes, including *APC*, *AXIN2*, *BMPR1A*, *GREM1*, *MUTYH*, *POLD1*, *POLE*, and *SMAD4.* Preliminary evidence genes could include *AKT1*, *BARD1*, *CDC73*, *CDH1*, *CHEK2*, *FAM175A*, *FANCC*, *MRE11A*, *MUTYH*, *PALB2*, *PIK3CA*, *POLD1*, *RAD50*, *RINT1*, *SDHB*, *SDHD*, and *XRCC2* when ovarian cancer is identified in the family and the colon research panel when uterine cancer is identified in the family.

### Genetic testing for classic cancer syndromes that include melanoma

Several cancer syndromes include a wide spectrum of cancers, including melanoma. Although, not all the syndromic manifestations are accounted for in our cancer assessment tool, suspicion should be raised when these features arise in a hereditary manner.

Patients with xeroderma pigmentosum (*XP* genes) have a greatly increased risk of non-melanoma skin cancer and a greater than 2000-fold risk of melanoma. [47]. Suspicion for this mutation should be raised in any child that develops cutaneous cancers within the first decade of life.

It is reasonable to suspect Lynch syndrome in any patient who develops melanoma in the context of the cancers arising from defects in the mismatch repair enzymes *MLH1*, *MSH2*, *EPCAM*, *MSH6*, and *PMS2*, including colon cancer and cancers of the endometrium, small intestine, ureter/uterus, or renal pelvis. Analysis of a Lynch syndrome registry has observed melanoma in patients with pathogenic mutations in these mismatch repair enzymes; however, the increased incidence was not found to be statistically significant [48]. Cowden Syndrome (CS) arises from a pathogenic mutation in the *PTEN* gene. CS is a multiple hamartoma syndrome with a high risk for benign and malignant tumors of the thyroid, breast, colon, and endometrium. Affected individuals usually have macrocephaly and a variety of skin lesions including trichilemmomas and papillomatous papules. Consensus diagnostic criteria for CS have been developed [49]. There is an evidence-based clinical scoring system available online to assist in selecting patients for genetics referral and *PTEN* testing [50]. The CS scoring system has been shown to be more accurate than the National Comprehensive Cancer Network (NCCN) diagnostic criteria [51]. The lifetime risk for cutaneous melanoma is estimated at more than 5%.

Germline mutation carriers of *TP53* have a substantial lifetime risk for a variety of cancers including childhood cancers and multiple primary cancers. Classically Li Fraumeni syndrome (LFS) families have a history of childhood leukemias, sarcomas, adrenal cortical carcinomas, and brain tumors and pre-menopausal breast cancers. Genetic testing should be considered for any individual diagnosed with a cancer at a younger age than normally expected or with multiple primary cancers. Increased rates of melanoma and non-melanoma skin cancers have been reported in families with LFS [52]. If a family seems to be falling into one of the classic cancer syndromes, then it makes sense to extend testing to other gene subsets reflexively if the initial testing is negative. Alternatively, if a particular cancer, such as melanoma, appears to be overrepresented, it would be reasonable to include the genes for that cancer in the panel.

## Alternative genetic testing strategies

A reasonable alternative to the tailored approach discussed above is to test for all known cancer predisposition genes, without discriminating based on the profile of cancers observed in the family. This approach may not be substantially more expensive and may detect genetic causes that were not expected based upon the cancers observed in the family. There are several large cancer panels available, but unfortunately, none of these include all the potential melanoma genes. Table 1 lists all of the currently known genes that have been strongly associated with a cancer predisposition syndrome, along with a designation for the cancer with which it has been associated. By including all of these genes in a single panel, every patient may be treated the same without risk of missing a candidate gene. However, with larger numbers of genes tested, the risk of an irrelevant mutation or VUS being identified will increase and complicate the genetic test reporting process.

Exome sequencing is another alternative approach. Although whole exome and whole genome sequencing is performed regularly on a research basis, the large numbers of variants of uncertain significance and the number of unanticipated actionable mutations make reporting extremely difficult. It is likely that these methods will eventually be applied to clinical genetic predisposition testing, but at present, it remains in the research realm for melanoma at our institutions.

## Tailored follow-up and management recommendations

Patients who have a family history concerning for a hereditary cancer syndrome or who have had an actionable mutation identified should be screened for associated cancers. In the USA, screening recommendations for mutation carriers are outlined in the NCCN Clinical Practical Guidelines in Oncology (NCCN Guidelines) or put forth by expert opinion consortiums. In the following paragraphs, we highlight these recommendations. These recommendations should be applied when one of these cancer types is seen in a melanoma-dominant or melanoma-subordinate syndrome and also when a gene is identified that is known to cause one of these syndromes.

### Melanoma screening recommendations

In melanoma-dominant or melanoma-subordinate pedigrees or in individuals carrying a *BAP1*, *CDK4*, *CDKN2A*, *MITF*, or *POT1* mutation, individuals should be educated on the importance of melanoma prevention and early detection. These individuals should be instructed on photoprotection and monthly self-skin screening examinations and should receive a regular skin screening examination by a medical professional. The frequency of examination by a health care provider should be tailored to account for the melanoma status and the difficulty of the examination, with higher-risk individuals receiving more frequent examinations ranging from every 3 to 12 months. If the individual has a personal history of melanoma, examinations should be in accordance with NCCN guidelines. If an individual has a large number of atypical nevi, it may be important to increase the frequency of appointments to 3–6 months to enhance surveillance. Children should begin self-screening as early as possible and provider-based screening should be initiated around the time of puberty. Monitoring these individuals with longitudinal photography and digital dermoscopy is helpful. A reduced threshold for biopsies of a suspicious lesion is reasonable in these patients. Annual screening for uveal melanoma, mesothelioma, and renal cancer should be considered for carriers of *BAP1* germline mutations [33].

### Breast and ovarian screening recommendations

Hereditary breast cancer genes can be classified in three categories. High-risk breast cancer genes are those associated with a fourfold relative risk. Moderate risk breast cancer genes are associated with a lifetime risk of twofold, with the risk being modifiable by family history. For the preliminary evidence breast cancer genes, the lifetime risk has not been defined, and therefore, estimation of risk is based solely on family history using the Claus tables or Tryer-Cuzick model.

The cornerstone of management is earlier and more frequent surveillance. For women who have high-risk breast cancer genetic mutations, breast awareness with self-breast exams and clinical breast exams is recommended starting at age 18. Imaging may begin as early as age 20 for *TP53* mutation carriers or age 25 for *BRCA1* and *BRCA2* mutation carriers. Because of the concern for lifetime radiation exposure associated with mammograms starting at such a young age, breast MRI is the imaging modality of choice between 20 and 30. Mammograms alternating with MRI with contrast every 6 months are recommended starting at age 30. Consideration of risk-reducing mastectomy (RRM) is an option for women who have mutations in risk genes conferring a 50% or greater lifetime risk for breast cancer or have a mutation in a moderate risk gene with significant family history.

If a woman has a mutation in a gene associated with an increased risk for ovarian cancer, the option of a salpingo-oophorectomy (RRSO) should be discussed between 35 and 40 years of age and upon completion of child bearing [53]. If a patient decides to forego RRSO, then transvaginal ultrasound and monitoring of CA-125 can be considered to monitor for ovarian cancer onset; however, this has not been shown to be sufficiently sensitive or specific to warrant recommendation.

### Prostate cancer screening recommendations

Guidelines for patients with a high-risk prostate cancer mutations have not been established by the NCCN; however, recent results from the IMPACT study have shown that the positive predictive values of prostate serum antigen (PSA) of 3.0 ng/mL are higher in patients with a high-risk genetic mutation compared to controls and that the former is more likely to have an intermediate or high-risk for disease [54]. They thus state it is reasonable to start with a baseline PSA and digital rectal exam (DRE) starting at age 40 rather than 45 years. If the DRE is normal and the PSA is less than1 ng/mL, then repeat testing should occur at 2–4 year intervals. If the DRE is normal and the PSA is between 1 and 3 ng/mL, then repeat testing should occur at 1–2 year intervals. If the PSA is greater than 3 ng/mL or the DRE is suspicious, then a TRUS-guided biopsy should be considered. An alternative to biopsy is to follow up in 6–12 months with a repeat PSA and DRE or determine the percent free PSA, 4Kscore, or PHI as detailed in their guidelines [55].

### Colon cancer screening recommendations

Generally, patients who have a high-risk colon cancer mutation should have a colonoscopy at age 20–25 years or 2 to 5 years prior to the earliest colon cancer if it is diagnosed before age 25 years. This should be repeated every 1 to 2 years [56]. There are, however, several more common high-risk hereditary colon cancer syndromes including Lynch syndrome, familial adenomatous polyposis (FAP), and *MUTYH*-associated polyposis (MAP) that have tailored follow-up.

Lynch syndrome is caused by autosomal dominantly inherited mutations in the mismatch repair genes *MLH1*, *MSH2*, *EPCAM*, *MSH6*, and *PMS2.* Individuals who carry mutations tend to form colon polyps at an earlier age and are at a high lifetime risk for colon cancer (up to 80%) unless frequent surveillance is done. Other associated cancer risks include uterine and ovarian cancer in female carriers and upper GI tumors, pancreatic cancer, urinary tract cancer, sebaceous neoplasms, and more rarely, brain cancer. Surveillance recommendations include colonoscopy starting at age 25 or 2–5 years prior to the earliest diagnosed colon cancer with follow-up every 1–2 years. There is no data to support screening for uterine or ovarian cancer. Women can consider a prophylactic hysterectomy and bilateral salpingo-oophorectomy after childbearing is complete. There is no evidence to support screening for upper GI tumors, but a baseline endoscopy at age 35 with follow-up every 3–5 years may be considered in select families (based on FH and ethnicity). An annual urinalysis can be done to screen for urinary tract cancers [56].

FAP is a colon cancer predisposition syndrome in which individuals develop hundreds to thousands of precancerous colonic polyps beginning, on average, at age 16 years (range 7–36 years). FAP is caused by autosomal dominantly inherited mutations in the *APC* gene, although 20–25% of probands have a *de novo* mutation. Ninety-five percent of individuals with classic FAP will develop colon cancer by age 35 unless a total colectomy is performed. Extracolonic manifestations may include polyps of the gastric fundus and duodenum, osteomas, dental anomalies, congenital hypertrophy of the retinal pigment epithelium (CHRPE), soft tissue tumors, desmoid tumors, and associated cancers. There is no known risk for melanoma in patients who have FAP.

Attenuated FAP is characterized by an increased risk for colon cancer but fewer colon polyps, more proximally located polyps, and diagnosis of colon cancer at a later age.

Gardner syndrome is characterized by polyposis typical of FAP together with osteomas and soft tissue tumors. Turcot syndrome is the association of colon polyps and central nervous system (CNS) tumors [57].

Patients who have classic FAP should have a proctocolectomy or total colectomy in early teenage years with follow-up surveillance of any remaining bowel every 1–3 years. Surveillance for thyroid cancer by annual thyroid exam stating in teenage years as well as upper endoscopy starting at age 20–25 is recommended. Patients who have attenuated familial adenomatous polyposis (AFAP) may be managed more conservatively with frequent colonoscopies if the polyp burden is manageable [56].

*MUTYH*-associated polyposis is caused by biallelic mutations in the *MUTYH* gene and tends to have a similar number of polyps as patients who have AFAP. Thus, genetic testing for both should be done in a patient who has greater than 20 polyps. Duodenal adenomas are found in 17–25% of individuals with MAP. Patients may also have serrated adenomas, hyperplastic/sessile serrated polyps, and mixed (hyperplastic and adenomatous) polyps. Frequency of colonoscopy and endoscopy depends on age and polyp load. A modestly increased risk for rather late-onset malignancies of the ovary, bladder, and skin is suspected, and there is some evidence for an increased risk for breast and endometrial cancer, but currently, there are no recommendations for surveillance [56].

### Pancreatic cancer screening recommendations

An international consortium panel released a consensus summary in 2013 on the management of high-risk individuals. There was general agreement that endoscopic ultrasonography (EUS) and/or MRI/magnetic resonance cholangiopancreatography should be utilized for initial screening. Screening is recommended for high-risk individuals, including patients with a family history of pancreatic cancer or patients with an *STK11*, *CDKN2A/p16*, or a *BRCA2* mutation with ≥1 affected first-degree relative. However, consensus was not reached for the age to initiate screening or to stop surveillance. At our institution, we start at 45 years of age or 10 years younger than the youngest diagnosed family member, whichever is younger. More evidence is needed, particularly for how to manage patients with detected lesions. Screening and subsequent management should take place at high-volume centers with multidisciplinary teams, preferably within research protocols [58].

### Other cancer screening recommendations

Melanoma syndromes have also been associated with uveal melanoma, renal cell carcinoma, astrocytoma, and other neurological cancers, mesothelioma and paraganglioma. Any pedigree that carries a mutation that overlaps with cancer predisposition genes for these syndromes or that has these types of cancers in the pedigree needs to be monitored carefully for signs and symptoms of these cancers. For example, ophthalmologic examinations should be performed every 6–12 months in *BAP1* mutation carriers. *BAP1* mutation carriers should also be explicitly warned about asbestos exposures, given their predisposition toward mesothelioma. Screening MRIs annually may be warranted if the pedigree or causative mutation is associated with multiple internal malignancies.

There are a larger number (variety) of cancers associated with Li Fraumeni including risks for childhood cancers. There are published guidelines for surveillance as well as significant controversy regarding the frequency of surveillance as well as the optimal modality [59].

## Conclusion

“Precision medicine” promises the use of an individual’s personal genome combined with a molecular analysis of that individual’s diseased tissue to identify rational therapies for that individual. The use of personal genomic information prior to the development of disease permits tailored surveillance recommendations, which may improve prevention and early detection, simultaneously reduce costs of care, and increase societal productivity. Our institutions have implemented panel testing for melanoma-dominant and melanoma-subordinate syndromes in order to provide state-of-the art care for these high-risk individuals as well as to promote further understanding of the spectrum of these syndromes.

The field of cancer genetics is quickly moving from a descriptive era, in which predisposition is defined primarily by disease phenotypes, into a whole-genome era, in which the relative contributions of high- and low-penetrance mutations, polymorphisms of modifier genes, and environmentally-induced somatic mutations are understood. During this transition period, patient care must be adjusted to keep pace with—and support further development of—the science of genetic predisposition.
